# *Origanum vulgare* and *Thymus vulgaris* Extract Usability to Improve Silage Hygienic Quality and Reduce Mycotoxin Concentrations

**DOI:** 10.4014/jmb.2003.03010

**Published:** 2020-05-21

**Authors:** Gintarė Vaičiulienė, Bronius Bakutis, Jurgita Jovaišienė, Rimvydas Falkauskas, Gediminas Gerulis, Violeta Baliukonienė

**Affiliations:** Department of Food Safety and Quality, Faculty of Veterinary Medicine, Veterinary Academy, Lithuanian University of Health Sciences, A. Mickevičiaus str. 9, LT-44307, Kaunas, Lithuania

**Keywords:** Herbal plants extracts, hygienic quality, mycotoxins, oregano, thyme, silage

## Abstract

Silage is one of the main feed components of ruminants around the world and can make up about 50- 80% of the rations of dairy cows during the winter. The aim of this study was to evaluate the usability of oregano (*Origanum vulgare* L.) and thyme (*Thymus vulgaris* L.) aqueous and ethanol extracts to improve the hygienic quality of perennial ryegrass, red clover and blue alfalfa silage samples and estimate their effect on mycotoxins concentrations. Under laboratory conditions, 63 silage samples (21 perennial ryegrass, 21 blue alfalfa, 21 red clover) were fermented with inserted aqueous and ethanol extracts of oregano and thyme and two commercial inoculants with mesophilic lactic acid bacteria. After 96 days of fermentation, in silage samples were established fermentation parameters, microbiological status and mycotoxins concentrations. It was determined that the best results for achieving hygienic quality of perennial ryegrass and red clover silage samples was by insertion of aqueous and ethanol extracts of oregano and thyme. In blue alfalfa samples, the best results of silage hygienic indicators were determined by inserting aqueous and ethanol extracts of oregano. Aflatoxin B1 (AFB1), deoxynivalenol (DON), zearalenone (ZEA) and T-2 toxin concentrations in perennial ryegrass, red clover and blue alfalfa silage samples were best reduced by inserting aqueous and ethanol extracts of oregano and thyme. The present study shows that these extracts can be used to improve silage hygienic quality, reduce mycotoxins concentrations and thus ensure the wellness of cattle.

## Introduction

As one of the main components of ruminant feed, silage can make up a large part of part of the diet of dairy cows during the winter [1]. Silage is obtained when grasses or other crops with sufficiently high moisture content are preserved by anaerobic fermentation [2]. In many countries, silage is a highly valued feed [3]. Production of high- quality silage depends on many controllable and uncontrollable factors, which can adversely affect silage production and utilization [4]. The most common measurements used for evaluating silage fermentation are pH, dry matter content and the sizes of various microbial populations [5]. Recent research has shown that among the most important and critical factors influencing silage hygienic quality are microbiological indicators such as the total number of aerobic and mesophilic lactic acid bacteria, counts of yeast and microscopic fungi spores and mycotoxin concentrations. Under aerobic conditions, yeast start to multiply and degrade lactic acid to produce heat. By the reduction of lactic acid, the pH-value increases. Consequently, other micro organisms, like molds and aerobic bacteria, get activated and cause the further spoilage of the silage [6].

Fungi are widespread. Commonly found in cattle feed are the microscopic fungi tribes of *Aspergillus* spp., *Penicillium* spp., *Fusarium* spp., *Claviceps* spp. and *Alternaria* spp., which at favorable conditions can produce secondary metabolites. Like many other agricultural products, silage can also be contaminated with these toxic metabolites, which can be produced in feeds through damage by technological ensiling processes. Currently, there are more than 400 known mycotoxins with about 30,000 derivatives. Among these, the most studied and most important in feed production are aflatoxins (AFs), deoxynivalenol (DON), zearalenone (ZEA) and T-2 toxin [7].

Mycotoxins are secondary metabolites with low molecular mass (~700 Da) and can cause mycotoxicosis [8, 9]. These compounds can have negative effects on animal health and productivity and can also cause adverse effects on human health [10]. For cattle, mycotoxins can cause carcinogenic, nephrotoxic and teratogenic effects, reproductive disorders (abortion, infertility), liver damage or immunosuppression [11].

Since ancient times the aromatic herbal plants have been used for their medicinal, aromatic and preservative properties [12]. Herbal extracts are concentrated preparations of a liquid consistency, usually obtained from dry (dried) phyto-raw material by extraction method. The objective of the extraction process is to maximize the amount of target compounds and to obtain the highest biological activity of these extracts. Many solvents, including methanol, ethanol, acetone or water, have been used for extracting bioactive compounds from plant materials. Due to the variety of bioactive compounds contained in plant materials and their differing solubility properties in different solvents, the optimal solvent for extraction depends on the particular plant materials and the compounds that are to be isolated [13]. So, in the production of oregano and thyme extracts as extraction solvents, due to their having the highest extraction yield, distilled water or various concentrations of ethanol are commonly used [14]. The main reason why herbs are used as antifungal agents is because they are natural and have little chance of developing pathogen resistance [15]. Antimicrobial activity in plant extracts is provided by secondary metabolites such as alkaloids, flavonoids, phenolic compounds and tannins [16]. In many representatives of oregano and thyme, oxygenated monoterpenes, thymol and carvacrol were found to be dominant components. The extracts have been assessed for antimicrobial effect and antioxidant activity against bacteria and microscopic fungi since they are generally recognized as safe (GRAS) by the United States Food and Drug Administration (FDA). Both substances appear to influence the cell membrane permeability causing the inhibition of growth and death of vegetative bacterial cells and also influence toxin production causing ion leakage and making microbes less virulent [17, 18]. Herbal plants extracts are widely used in medicine, animal breeding and food areas, also as alternative food additives to protect against foodborne pathogens and to reduce food or feed spoilage [19].

The aim of this study was to evaluate the usability of oregano (*Origanum vulgare* L.) and thyme (*Thymus vulgaris* L.) aqueous and ethanol extracts to improve the hygienic quality of perennial ryegrass, red clover and blue alfalfa silage and estimate their effect on mycotoxin concentrations.

## Materials and Methods

### Plant Materials

Perennial ryegrass (*Phleum pretense*, *Lolium perenne*, *Festuca pratensis*), blue alfalfa (*Medicago sativa* L.) and red clover (*Trifolium pretense*) were collected from three different farms in Lithuania for silage production under laboratory conditions. Aqueous and ethanol extracts were prepared under laboratory conditions from oregano (*O. vulgare* L.) and thyme (*T. vulgaris* L.) herbal plant materials. The air-dried plant materials were purchased from the Lithuanian company “Švenčionių vaistažolės.”

### Preparation of *O. vulgare* and *T. vulgaris* Aqueous and Ethanol Extracts

The extracts were prepared by maceration using water and ethanol as solvents following Handa *et al*. [20] and Tayel *et al*. [21] techniques. Twenty-five grams of dried, chopped individual herbal plant mass was mixed with 200 ml of distilled water or 200 ml 70% ethanol, covered, and stored for 24 h at room temperature, avoiding direct solar radiation. After 24 h, the solutions were filtered using Whatman no. 1 filter paper. The obtained extracts were inserted at once into the ensiled perennial ryegrass, blue alfalfa and red clover silage samples.

### Determination of Monoterpenoid Phenols (Carvacrol, Thymol) in Herbal Plant Extracts

Monoterpenoid phenols (carvacrol, thymol) in oregano and thyme aqueous and ethanol extracts were determined by high-performance liquid chromatography (HPLC) (Agilent 1260 Infinity, USA) In State Research Institute, Centre for Physical Sciences and Technology, Vilnius, Lithuania. Monoterpenoid phenol absorbance was determined on an ultraviolet (UV) background at 274 nm. The detection limit of carvarcol was 2.5 μg/ml and thymol – 2.4 μg/ml. Sample preparation was repeated three times.

### Preparation of Silage Samples with Inserted Herbal Plant Extracts Under Laboratory Conditions

Perennial ryegrass, blue alfalfa and red clover were chosen as the raw materials for silage samples. The harvested and chopped raw mass was ensiled in 3 L mini-silos: 20 ml of each herbal plant extract and 1 kg of each raw material separately were well mixed and compressed into the mini silos. Preparation of plant extracts: Thyme aqueous extract (T-AE)-25 – 20 ml aqueous extract of *Thymi herba* (25 g of *T. herba* and 200 ml of distilled water); Thyme ethanol extract (T-EE)-25 – 20 ml ethanol extract of *T. herba* (25 g of *T. herba* and 200 ml 70% of ethanol); Oregano aqueous extract (O-AE)-25 – 20 ml aqueous extract of *Origani herba* (25 g of *O. herba* and 200 ml of distilled water); Oregano ethanol extract (O-EE)-25 – 20 ml ethanol extract of *O. herba* (25 g of *O. herba* and 200 ml of 70% ethanol). Also produced was silage with two commercial additives (silage inoculants) according to the manufacturer's instructions. The additives contained the following bacteria: additive X – *L. plantarum*, *E. faecium*, *P. acidilactici* (1.0 × 10^11^ CFU/g) and additive Y – *L. plantarum*, *P. acidilactici* (1.0 × 1011 CFU/g) and xylanase. The same principle was used for production of the control silage (C) without inserting anything. The silos were hermetically closed and stored for 96 days at 20°C. For each extract and control, three mini silos were made. Ensiling under laboratory conditions was conducted based on modified Tayel *et al*. [22] and Jatkauskas *et al.* [23] methods. After 96 days of fermentation, silos were opened and 63 silage samples (*n* = 21 perennial ryegrass, *n* = 21 blue alfalfa, *n* = 21 red clover) were taken for assessment by sensory evaluation, fermentation parameters, microbiological status and mycotoxin concentrations.

### Determination of Dry Matter (DM) Content and pH

The dry matter content was analyzed by the weight method. In order to determine dry matter, the silage samples were chopped into 4-cm diameter particles and dried for 18 h at 55°C. After air equilibration, samples were weighed and then dried again for 20 h at 103°C [24]. The level of pH was measured by electrometric method in diluted silage with a pH-meter (WTW inoLab pH 720, Germany) fitted with a glass electrode after homogenization of 10 g silage sample with 40 ml of distilled water. Once a constant value was found, the pH was determined from the dial reading with an accuracy of at least 0.05 pH unit.

### Determination of Microbiological Indicators

Microbiological indicators of silage samples were defined according to standards: total count of aerobic bacteria (LT ISO 4833-1:2013), total count of mesophilic lactic acid bacteria (LT ISO 15214:2009) and total count of yeast and molds (LT ISO 21527-1:2008).

### Determination of Mycotoxins Concentrations by ELISA Assay

The mycotoxins concentrations in samples were determined by direct competitive enzyme-linked immunosorbent assays (ELISA) [24]. Contamination with aflatoxin B1 (AFB1), deoxynivalenol (DON), zearalenone (ZEA) and T-2 toxin was tested. The VERATOX test kits (Neogen Corporation, Scotland) approved by the AOAC Research Institute (Certificate No. 950702) were used for the analysis. Mycotoxin extraction and tests were performed according to manufacturer’s recommendations. Whole perennial ryegrass, blue alfalfa and red clover samples were air-dried, ground to pass a 1 mm screen and homogenized. Extraction of samples was carried out in distilled water for DON, in methanol:water (70:30 v/v) for AFB1 and ZEA. Absorbance was determined using the microwell strip reader (Bio-tek Synergy HT, USA) at 650 nm. A calibration curve for the standards for each AFB1, ZEA, DON, T-2 toxin dilution was plotted using a standard concentration against the percentage inhibition of the respective standard. For determination, each mycotoxin concentration was automatically calculated from the calibration curves, obtained by plotting absorbance intensity against the logarithm of analytic concentration. The measured absorbance was automatically converted to the mycotoxin concentration units – μg/kg. The estimation took into account the lowest calibration curve’s mycotoxin concentration value (LOD - limit of detection), which is for AFB1 – 2.0 μg/kg (ppb), ZEA – 10.0 μg/kg (ppb), DON – 100.0 μg/kg (ppb) and T-2 toxin – 10.0 μg/kg (ppb) (according to the manufacturer’s guidelines).

### Statistical Analysis

The mean value of the results was expressed of at least 3 measurements ± standard deviation (SD). To evaluate the effect of the herbal plant extracts and commercial inoculants on perennial ryegrass, red clover and blue alfalfa silage hygienic quality and mycotoxin concentrations, the data were analyzed by descriptive statistic and one-way ANOVA (SPSS version 20.0, IBM Corp., USA). The results were considered to be statistically significant at *p* ≤ 0.05.

## Results

### Quantitative Analysis of Monoterpenoid Phenols (Carvacrol, Thymol) in *O. vulgare* and *T. vulgaris* Aqueous and Ethanol Extracts

Carvacrol and thymol concentrations in oregano and thyme aqueous and ethanol extracts are presented in Table 1. The highest concentrations of carvacrol and thymol were established in ethanol extracts of oregano and thyme. These phenolic compounds were detected neither in oregano nor thyme aqueous extracts.

### Effect of *O. vulgare* and *T. vulgaris* Aqueous and Ethanol Extracts on Silage Hygienic Quality After 96 Days of Fermentation

Silage samples after 96 days of fermentation were assessed by sensory evaluation. All perennial ryegrass, red clover and blue alfalfa silage samples were evaluated by senses: color, odor, texture, and absence or presence of molds. All of the samples met the requirements for good quality silage.

The hygienic quality of ryegrass, red clover and blue alfalfa silage samples after 96 days of fermentation is presented in Table 2. In the analyzed perennial ryegrass, red clover and blue alfalfa silage samples, pH varied from 3.68 to 6.68, the dry matter content – from 21-57%. For estimated hygienic quality of perennial ryegrass silage samples, the best results were obtained with inserted oregano aqueous and thyme ethanol extracts. Of all inserted herbal plant extracts and commercial inoculants, these extracts most effectively reduced total count of aerobic bacteria and count of yeast and molds. In perennial ryegrass silage samples with inserted oregano aqueous and thyme ethanol extracts, count of yeast and molds weren’t detected. While the highest count of mesophilic lactic acid bacteria was established with inserted oregano ethanol extract. Among all red clover silage samples with inserted herbal plant extracts and commercial inoculants, the best results and the lowest total count of aerobic bacteria were determined in red clover silage sample with inserted thyme ethanol extract (1.55 ± 0.12 log10 CFU/g). The lowest count of yeast was established with inserted oregano aqueous extract (0.94 ± 0.03 log10 CFU/g), which was 53.9%, respectively, lower compared to the control red clover silage sample (1.92 ± 0.02 log10 CFU/g). Red clover silage samples with inserted oregano and thyme aqueous extracts and commercial inoculants X showed the best results and reduced count of molds by 100%, compared to the control silage samples. Meanwhile, the highest total count of mesophilic lactic acid bacteria was established in control red clover silage sample, without inserting anything. In the all analyzed samples, oregano aqueous extract inserted in blue alfalfa silage showed the best results and in this sample was determined the lowest total count of aerobic bacteria (1.26 ± 0.08 log10 CFU/g). Total count of yeast and molds in blue alfalfa silage was reduced by 100%, compared to the control samples with inserted oregano aqueous and ethanol extracts and commercial inoculant X. Also the highest total count of mesophilic lactic acid bacteria was determined with inserted thyme aqueous extract (2.05 ± 0.01 log10 CFU/g), which was 10.4%, respectively, higher compared to the control blue alfalfa silage (1.84 ± 0.03 log10 CFU/g).

### Effect of *O. vulgare* and *T. vulgaris* Aqueous and Ethanol Extracts on Mycotoxins AFB1, ZEA, DON, and T-2 Toxin Concentrations After 96 Days of Fermentation

Table 3 reveals the concentrations of mycotoxins AFB1, ZEA, DON and T-2 toxin in the perennial ryegrass, red clover and blue alfalfa silage samples with inserted herbal plants extract and commercial inoculants with mesophilic lactic acid bacteria. A very low quantity of AFB1 concentration was detected in all estimated perennial ryegrass, red clover and blue alfalfa silage samples, and in most of them AFB1 concentration was below the detection limit. The highest AFB1 concentrations were established in red clover silage with inserted oregano ethanol extract (4.99 ± 0.02 μg/kg) and blue alfalfa silage with inserted commercial inoculant Y (7.00 ± 0.17 μg/kg). In perennial ryegrass silage with inserted thyme ethanol extract (130 ± 0.00 μg/kg) was detected the lowest ZEA concentration, which was 74.0%, lower compared to the control perennial silage sample (500 ± 0.00 μg/kg). The lowest DON concentration (300 ± 0.00 μg/kg) was established in silage samples with inserted commercial inoculants X and Y, which were 40%, respectively, lower than in the control silage sample (500 ± 10.00 μg/kg). The strongest effect on T-2 toxin concentration reduction in perennial ryegrass silage was shown by inserted thyme ethanol extract, which decreased T-2 toxin concentration by 100%, compared to the control silage. Among all red clover silage samples with inserted herbal plants extracts and commercial inoculants, the best results of ZEA concentration reduction were obtained with inserted oregano aqueous extract (300 ± 10.00 μg/kg), which was 75.0%, respectively, lower than in the control red clover silage sample (1200 ± 50.00 μg/kg). The lowest DON concentration was found in red clover silage with inserted oregano aqueous extract (200 ± 10.00 μg/kg), which was 55.5%, lower compared to the control silage (450 ± 10.00 μg/kg). In 71.4% of all red clover samples T-2 toxin concentration was below the detection limit, and the best results were obtained with inserted oregano aqueous and ethanol extracts, thyme ethanol extract and commercial inoculants X and Y. Different tendencies of ZEA, DON and T-2 toxin concentrations in blue alfalfa samples were found. The best result and the lowest ZEA concentration was detected in silage sample with inserted oregano ethanol extract (150 ± 0.00 μg/kg), which was 44.4% lower, compared to the control blue alfalfa silage (270 ± 10.00 μg/kg). Among all blue alfalfa samples with inserted herbal plants extracts and commercial inoculants, the lowest DON concentration was established in the blue alfalfa silage with inserted thyme aqueous extract and commercial inoculant X (150 ± 10.00 μg/kg), whereas in control blue alfalfa silage sample (550 ± 10.00 μg/kg), concentration was 72.7% higher. The strongest effect on T-2 toxin concentration reduction in blue alfalfa silage was shown by inserted oregano and thyme ethanol extracts, which decreased T-2 toxin concentration by 100%, compared to the control silage sample.

## Discussion

Infection of silage with potentially pathogenic microorganisms is a growing problem not only in Europe but also worldwide. It causes enormous economic losses for livestock farmers and has a negative impact on animal health [25, 26]. For this reason, scientists are currently looking for the best, safest, natural and biologically active substances to improve feed fermentation and ensure the sanitary hygienic indicators [22]. Usually silage is ensiled from maize, grass, clover, sugar beet tops, alfalfa or milo [27]. Until now, most researchers focused on grains, cereals and maize silage hygienic quality and mycotoxins occurrence while fewer studies were done with grass silage and legumes. For this reason, in our experiment we chose perennial ryegrass, red clover and blue alfalfa silage in order to evaluate their fermentability differences and the effects of inserted herbal plant extracts on hygienic quality and mycotoxin concentrations. It has been proved that plant extracts obtained with different solvents and essential oils are rich in potentially bioactive compounds, which gives the ability to attack different fungal genera and hinders the development of resistance by the pathogen [28].

Plants in the family *Lamiaceae* have been drawing huge attention from the scientific community because of their antioxidant and antimicrobial properties [29, 30]. According to studies, extracts of this plant family containing phenolic compounds can influence feed epiphytic microflora (fungi, yeast) inhibiting the integrity of the cell membrane, thus changing inorganic ion balance and pH homeostasis. [31]. Carvacrol and thymol are the two main phenols, accounting for about 78 to 85% of oregano essential oils. Essential oil of thyme contains thymol (44.0-60.0%) and carvacrol (2.2-4.2%) [32]. Analysis of scientific literature results has shown that different solvents resulted in various extraction yields. This is because differences in the polarity of the extraction solvents could cause a wide variation in the level of bioactive compounds in the extract and the highest extraction yield was observed with ethanol and distilled water extracts [13]. We suggest that this could be because the plant materials contain high levels of polar compounds that are soluble in solvents with high polarity such as water, methanol or ethanol. In order to better understand the solvents effect and evaluate their antimicrobial activity and effect on mycotoxin concentrations, we also have chosen aqueous and ethanol extracts of thyme and oregano, which were incorporated into the green mass of plants before ensiling. In the present study, the highest concentrations of the main active components of *O. vulgar*e and *T. vulgaris*, carvacrol and thymol, were established in oregano (respectively 5.3 ± 2.9%, 8.5 ± 0.7%) and thyme (94.5 ± 0.3%, 81.9 ± 0.8%) ethanol extracts. Gallucci and co-authors [33] have found that carvacrol, eugenol, linalool, cinnamon aldehydes and thymol have strong antimicrobial activity against many microorganisms. From our data we can also state that the best hygienic quality of perennial ryegrass and red clover silage was determined with inserted oregano aqueous and thyme ethanol extracts. These extracts inserted in red clover silage samples effectively reduced total count of aerobic bacteria, mesophilic lactic acid bacteria, and count of yeast and molds (*p* ≤ 0.05). Inserted in perennial ryegrass silage, the extracts reduced total count of mesophilic lactic acid bacteria and count of yeast and molds (*p* ≤ 0.05). While in blue alfalfa silage, the same results were achieved with inserted oregano extracts made with different solvents (*p* ≤ 0.05).

Mycotoxins are toxic secondary metabolites of microscopic fungi, which for cattle can reduce production output, weaken the immune system, cause damage to the liver, kidneys, digestive tract and organs of the reproductive system [25, 26]. There is still a lack of information on methods for application of the plant extracts to effectively prevent the fungal development. However, fumigation is one of the best methods to protect feed or feed components against contamination during storage. Plant extracts fumigation has already been considered an eco- friendly alternative to prevent fungal growth and the production of mycotoxins [34]. Based on our analysis of different botanical composition silage samples, AFB1 concentration varied from below detection limit to 7.0 μg/kg, ZEA – 130-1300 μg/kg, DON – 150-550 μg/kg and T-2 toxin from below detection limit to 250 μg/kg. According to the authors, AFB1 is not considered a major problem in many European countries, increasingly and in larger quantities in silage feeds are determined ZEA and DON mycotoxins. In the present study, the highest ZEA and DON concentrations were determined in analyzed silage samples, while AFB1 concentration in most of samples was established below detection limit. Based on our research, thyme ethanol extract inserted in perennial ryegrass silage reduced AFB1, ZEA and T-2 toxin concentrations (*p* ≤ 0.05), while concentration of DON (*p* ≤ 0.05) was best reduced with thyme aqueous extract. The lowest AFB1 (*p* ≤ 0.05) and T-2 toxin (*p* ≥ 0.05) concentrations in red clover silage were determined with inserted oregano and thyme ethanol extracts, while for ZEA and DON concentrations (*p* ≤ 0.05) the lowest was with inserted oregano aqueous extract. AFB1 and ZEA concentrations (*p* ≤ 0.05) in blue alfalfa silage were reduced with inserted oregano aqueous and ethanol extracts, while concentrations of DON and T-2 toxin (*p* ≤ 0.05) were reduced with inserted thyme aqueous and thyme and oregano ethanol extracts. Research by us and other authors indicates that antimicrobial potential of thymol and carvacol (monoterpenoid phenols from *O. vulgare* and *T. vulgaris*) can prevent the formation of mycotoxin concentrations in silo.

In conclusion, after evaluating silage samples with and without herbal plant extracts and commercial inoculants, we have determined that the best results for hygienic quality of perennial ryegrass and red clover silage samples were established with inserted oregano aqueous and thyme ethanol extracts. While in blue alfalfa samples, the best results of silage hygienic indicators were determined with inserted oregano aqueous and ethanol extracts. Based on the obtained research studies, our findings showed, that aqueous and ethanol extracts of oregano and thyme reduced mycotoxins AFB1, DON, ZEA, and T-2 toxin concentrations in perennial ryegrass, red clover and blue alfalfa silage samples. Additionally, further detailed studies are necessary to identify the main active compounds of plant extracts. Especially, further studies are required with the main active substances of *O. vulgare* and *T. vulgaris*, carvacrol and thymol, to investigate their mechanisms of action in silage samples. Our data indicate that *O. vulgare* and *T. vulgaris* aqueous and ethanol extracts can be very useful in silage production practices to improve silage hygienic quality and reduce mycotoxins concentrations.

## Figures and Tables

**Table 1:** Concentrations (%) of carvacrol and thymol of herbal plants extracts.

Analyte, µg/ml	Oregano aqueous extract (O-AE)	Oregano ethanol extract (O-EE)	Thyme aqueous extract (T-AE)	Thyme ethanol extract (T-EE)
Carvacrol ± SD, %	LOD	5.3 ± 2.9	LOD	94.5 ± 0.3
Thymol ± SD, %	LOD	8.5 ± 0.7	LOQ	81.9 ± 0.8

SD – standard deviation (%)

LOD – limit of detection, LOQ - limit of quantification

**Table 2:** Perennial ryegrass, red clover and blue alfalfa silage hygienic quality after 96 days of fermentation.

Silage samples	DM ± SD, %	pH ± SD	Total count of aerobic bacteria ± SD, log_10_ CFU/g	Count of yeast ± SD, log_10_ CFU/g	Count of molds ± SD, log_10_CFU/g	Total count of mesophilic lactic acid bacteria ± SD, log_10_ CFU/g
Perennial ryegrass C	20.66 ± 1.52^b,c,d,e,f,g^	6.11 ± 0.01^b,c,d,e,f,,g^	2.36 ± 0.17	1.79 ± 0.02^b,c,d,e,f,g^	0.84 ± 0.03^b,c,d,e,f,g^	1.91 ± 0.01^b,c,e,g^
Perennial ryegrass O-AE	32.66 ± 4.50^a,f^	6.68 ± 0.02^a,c,d,e,f,g^	2.48 ± 0.65	0.00 ± 0.00^a,c,d,f,g^	0.00 ± 0.00^a,c,f,g^	1.74 ± 0.02^a,c,d,f,g^
Perennial ryegrass O-EE	33.00 ± 1.00^a,f^	4.82 ± 0.02^a,b,d,e,f,g^	2.64 ± 0.30	1.45 ± 0.04^a,b,d,e,f,g^	0.76 ± 0.02^a,b,d,e,f,g^	2.04 ± 0.00^a,b,d,e,f,g^
Perennial ryegrass T-AE	30.66 ± 0.57^a,f,g^	5.05 ± 0.02^a,b,c,e,f,g^	2.81 ± 0.04	1.14 ± 0.01^a,b,c,e,g^	0.00 ± 0.00^a,c,f,g^	1.88 ± 0.01^b,c,e,g^
Perennial ryegrass T-EE	32.00 ± 2.64^a,f^	4.35 ± 0.01^a,b,c,d,f,g^	2.49 ± 0.04	0.00 ± 0.00^a,c,d,f,g^	0.00 ± 0.00^a,c,f,g^	1.74 ± 0.03^a,c,d,f,g^
Perennial ryegrass X	37.00 ± 1.00^a,b,c,d,e^	4.47 ± 0.02^a,b,c,d,e^	2.49 ± 0.25	1.17 ± 0.04^a,b,c,e,g^	0.47 ± 0.05^a,b,c,d,e,g^	1.88 ± 0.02^b,c,e,g^
Perennial ryegrass Y	35.66 ± 0.57^a,d^	4.48 ± 0.02^a,b,c,d,e^	2.30 ± 0.01	0.60 ± 0.03^a,b,c,d,e,f^	1.08 ± 0.03^a,b,c,d,e,f^	1.80 ± 0.01^a,b,c,d,e,f^
						
Red clover C	29.66 ± 1.52^y,k,l,m^	4.36 ± 0.02^i,y,j,k,l,m^	2.37 ± 0.10^i,j,k,l,m^	1.92 ± 0.02^i,y,j,k,l^	0.63 ± 0.04^i,j,k,l,m^	2.35 ± 0.01^i,y,j,k,l,m^
Red clover O-AE	30.33 ± 1.52^y,j,k,l,m^	4.64 ± 0.01^h,y,j,k,l,m^	2.00 ± 0.01^h,y,j,k,l^	0.95 ± 0.03^h,y,j,k,l,m^	0.00 ± 0.00^h,y,k,m^	1.92 ± 0.02^h,y,j,k,l,m^
Red clover O-EE	38.00 ± 2.00^h,i,j,k,l^	4.13 ± 0.04^h,i,j,k,l^	2.33 ± 0.05^i,j,k,l,m^	1.78 ± 0.04^h,i,j,k,l,m^	0.60 ± 0.02^i,j,k,l,m^	1.23 ± 0.01^h,i,j,k,l,m^
Red clover T-AE	27.66 ± 1.52^i,y,k,l,m^	5.05 ± 0.02^h,i,y,k,l,m^	1.79 ± 0.02^h,i,y,k,l,m^	1.56 ± 0.01^h,i,y,k,l,m^	0.00 ± 0.00^h,y,k,m^	1.77 ± 0.01^h,i,y,k,l,m^
Red clover T-EE	56.66 ± 1.15^h,i,y,j,l,m^	5.43 ± 0.03^h,i,y,j,l,m^	1.55 ± 0.12^h,i,y,j,l,m^	1.22 ± 0.04^h,i,y,j,l,m^	0.77 ± 0.01^h,i,y,j,l^	1.64 ± 0.02^h,i,y,j,l,m^
Red clover X	53.66 ± 1.52^h,i,y,j,k,m^	4.31 ± 0.01^h,i,y,j,k,m^	2.14 ± 0.05^h,i,y,j,k,m^	0.84 ± 0.06^h,i,y,j,k,m^	0.00 ± 0.00^h,y,k,m^	1.45 ± 0.03^h,i,y,j,k,m^
Red clover Y	38.66 ± 0.57^h,i,j,k,l^	4.08 ± 0.01^h,i,j,k,l^	2.01 ± 0.01 ^h,y,j,k,l^	1.86 ± 0.01^i,y,j,k,l^	0.77 ± 0.02^h,i,y,j,l^	1.41 ± 0.04^h,i,y,j,k,l^
						
Blue alfalfa C	26.66 ± 1.52^o,p,r,s,t,u^	4.90 ± 0.05^o,p,r,s,t,u^	1.65 ± 0.09^o,r,s,t,u^	0.59 ± 0.01^o,p,r,s,t,u^	0.30 ± 0.01^o,p,r,s,t,u^	1.84 ± 0.03^o,p,r,s,t,u^
Blue alfalfa O-AE	55.66 ± 1.15^n,p,r,s,u^	4.30 ± 0.02^n,p,r,s,t,u^	1.26 ± 0.08^n,p,r,s,t,u^	0.00 ± 0.00^n,r,s,u^	0.00 ± 0.00^n,r,s^	1.30 ± 0.01^n,p,r,s,t,u^
Blue alfalfa O-EE	49.66 ± 1.15^n,o,r,s,t,u^	4.42 ± 0.02^n,o,r,s,t,u^	1.69 ± 0.04^o,r,s,t,u^	0.00 ± 0.00^c,r,s,u^	0.00 ± 0.00^n,r,s^	1.36 ± 0.01^n,o,r,s,t,u^
Blue alfalfa T-AE	36.66 ± 0.57^n,o,p,t,u^	4.52 ± 0.02^n,o,p,s,t,u^	1.87 ± 0.01^n,o,p,s,t,u^	0.46 ±0.02^n,o,p,s,t,u^	0.69 ± 0.02^n,o,p,s,t,u^	2.05 ± 0.01^n,o,p,s,t,u^
Blue alfalfa T-EE	36.33 ± 1.52^n,o,p,t,u^	3.88 ± 0.01^n,o,p,r,t,u^	2.00 ± 0.01^n,o,p,r,t^	1.11 ± 0.01^n,o,p,r,t^	1.14 ± 0.05^n,o,p,r,t,u^	1.73 ± 0.03^n,o,p,r,t,u^
Blue alfalfa X	53.00 ± 1.73^n,p,r,s,u^	5.27 ± 0.02^n,o,p,r,s,u^	1.49 ± 0.08^n,o,p,r,s,u^	0.00 ± 0.00^n,r,s,u^	0.00 ± 0.00^n,r,s^	1.49 ± 0.02^n,o,p,r,s,u^
Blue alfalfa Y	45.66 ± 2.88^n,o,p,r,s,t^	3.67 ± 0.01^n,o,p,r,s,t^	2.06 ± 0.03^n,o,p,r,u^	1.11 ±0.07^n,o,p,r,t^	0.00 ± 0.00^n,r,s^	1.97 ± 0.01^n,o,p,r,s,t^

^a_g^Mean values with different superscripts are significantly different in perennial ryegrass silage samples (*p* ≤ 0.05). ^h_m^Mean values with different superscripts are significantly different in red clover silage samples (*p* ≤ 0.05). ^n_u^Mean values with different superscripts are significantly different in blue alfalfa silage samples (*p* ≤ 0.05).

SD – standard deviation

C – control sample, O-AE – oregano aqueous extract, O-EE – oregano ethanol extract, T-AE – thyme aqueous extract, T-EE –thyme ethanol extract, X – commercial inoculant X, Y – commercial inoculant Y.

**Table 3:** Mycotoxin concentrations in perennial ryegrass, red clover and blue alfalfa silage after 96 days of fermentation.

Silage samples	Mycotoxins concentrations ± SD, µg/kg (ppb)			

	AFB1	ZEA	DON	T-2
Perennial ryegrass C	1.67 ± 0.06^b,c,d,e,f,g^	500 ± 0.00^b,c,d,e,f,g^	500 ± 10.00^b,c,d,e,f,g^	110 ±10.00^b,c,d,e,f,g^
Perennial ryegrass O-AE	0.99 ± 0.01^a,c,d,f,g^	170 ± 17.32^a,c,d,e,f,g^	550 ± 0.00^a,c,d,e,f,g^	80 ± 5.00^a,c,d,e,f^
Perennial ryegrass O-EE	0.70 ± 0.03^a,b,d,e,f^	300 ± 0.00^a,b,e,f,g^	400 ± 10.00^a,b,e,f,g,^	200 ± 0.00^a,b,d,e,f,g^
Perennial ryegrass T-AE	n.d	300 ± 10.00^a,b,e,f,g^	400 ± 10.00^a,b,e,f,g^	150 ± 5.00^a,b,c,e,f,g^
Perennial ryegrass T-EE	0.99 ± 0.04^a,c,d,f,g^	130 ± 0.00^a,b,c,d,f,g^	450 ± 20.00^a,b,c,d,f,g^	n.d
Perennial ryegrass X	n.d	670 ± 10.00^a,b,c,d,e,g^	300 ± 0.00^a,b,c,d,e^	125 ± 5.00^a,b,c,d,e,g^
Perennial ryegrass Y	0.70 ± 0.01^a,b,d,e,f^	200 ± 5.00^a,b,c,d,e,f^	300 ± 10.00^a,b,c,d,e^	75 ± 8.66^a,c,d,e,f^
				
Red clover C	2.00 ± 0.01^y,j,k,l,m^	1200 ± 50.00^i,y,j,k,l,m^	450 ± 10.00^i,y,j,k,l,m^	n.d
Red clover O-AE	2.00 ± 0.02^y,j,k,l,m^		300 ± 10.00^h,y,j,k,l,m^	200 ± 10.00^h,y,j,k,l,m^
Red clover O-EE	4.99 ± 0.02^h,i,j,k,l,m^	600 ± 000^h,i,y,j,l,m^	500 ± 10.00^h,i,k,l^	n.d
Red clover T-AE	n.d	700 ± 10.00^h,i,y,l,m^	500 ± 0.00^h,i,k,l^	25 ± 5.00^h,i,y,k,l,m^
Red clover T-EE	3.01 ± 0.02^h,i,y,j,l,m^	670 ± 8.66^h,i,l,m^	350 ± 10.00^h,i,y,j,k,m^	n.d
Red clover X	n.d	470 ± 10.00^h,i,y,j,k,m^	350 ± 0.00^h,i,y,j,m^	n.d
Red clover Y	n.d	1300 ± 100.00^h,i,y,j,k,l^	500 ± 10.00^h,i,k,l^	n.d
				
Blue alfalfa C	0.73 ± 0.04^o,p,r,s,t,u^	270 ± 10.00^o,p,r,s,t,u^	550 ± 10.00^o,p,r,s,t,u^	50 ± 5.00^o,p,r,s,t,u^
Blue alfalfa O-AE	0.70 ± 0.01^p,r,s,t,u^	170 ± 10.00^n,r,s,t,u^	500 ± 0.00^n,r,t,u^	75 ± 5.00^n,p,r,s,t,u^
Blue alfalfa O-EE	3.00 ± 0.10^n,o,r,t,u^	150 ± 0.00^n,r,s,t,u^	500 ± 10.00^n,r,t,u^	n.d
Blue alfalfa T-AE	2.67 ± 0.12^n,o,p,s,t,u^	470 ± 10.00^n,o,p,s,u^	150 ± 10.00^n,o,p,r,s,u^	150 ± 10.00^n,o,p,s,u^
Blue alfalfa T-EE	3.03 ± 0.06^n,o,r,t,u^	200 ± 10.00^n,o,p,r,s,t,u^	500 ± 0.00^n,r,t,u^	n.d
Blue alfalfa X	1.30 ± 0.20^n,o,p,r,s,u^	470 ± 20.00^n,o,p,r,s,u^	150 ± 10.00^n,o,p,r,s,u^	150 ± 0.00^n,o,p,s,u^
Blue alfalfa Y	7.00 ± 0.17^n,o,p,r,s,t^	680 ± 8.66^n,o,p,r,s,^t	250 ± 20.00^n,o,p,r,s,t^	250 ± 26.46^n,o,p,r,s,t^

* n.d. – not detected

SD – standard deviation

^a – g^ Mean values with different superscripts are significantly different in perennial ryegrass silage samples (*p* ≤ 0.05). ^h – m^ Mean values with different superscripts are significantly different in red clover silage samples (*p* ≤ 0.05). ^n – u^ Mean values with different superscripts are significantly different in blue alfalfa silage samples (*p* ≤ 0.05).

C – control sample, O-AE – oregano aqueous extract, O-EE – oregano ethanol extract, T-AE – thyme aqueous extract, T-EE – thyme ethanol extract, X – commercial inoculant X, Y – commercial inoculant Y.
